# Phase I Trial of ^99m^Tc-(HE)_3_-G3, a DARPin-Based Probe for Imaging of HER2 Expression in Breast Cancer

**DOI:** 10.2967/jnumed.121.262542

**Published:** 2022-04

**Authors:** Olga Bragina, Vladimir Chernov, Alexey Schulga, Elena Konovalova, Eugeniy Garbukov, Anzhelika Vorobyeva, Anna Orlova, Liubov Tashireva, Jens Sörensen, Roman Zelchan, Anna Medvedeva, Sergey Deyev, Vladimir Tolmachev

**Affiliations:** 1 Department of Nuclear Medicine, Cancer Research Institute, Tomsk National Research Medical Center, Russian Academy of Sciences, Tomsk, Russia;; 2 Research Centrum for Oncotheranostics, Research School of Chemistry and Applied Biomedical Sciences, Tomsk Polytechnic University, Tomsk, Russia;; 3 Shemyakin-Ovchinnikov Institute of Bioorganic Chemistry of the Russian Academy of Sciences, Moscow, Russia;; 4 Department of General Oncology, Cancer Research Institute, Tomsk National Research Medical Center Russian Academy of Sciences, Tomsk, Russia;; 5 Department of Immunology, Genetics and Pathology, Uppsala University, Uppsala, Sweden;; 6 Department of Medicinal Chemistry, Uppsala University, Uppsala, Sweden;; 7 Department of General and Molecular Pathology, Tomsk National Research Medical Center, Tomsk, Russia; and; 8 Radiology and Nuclear Medicine, Department of Surgical Sciences, Uppsala University, Uppsala, Sweden

**Keywords:** HER2, DARPin G3, ^99m^Tc, SPECT, phase I

## Abstract

Radionuclide molecular imaging of human epidermal growth factor receptor type 2 (HER2) expression may enable a noninvasive discrimination between HER2-positive and HER2-negative breast cancers for stratification of patients for HER2-targeted treatments. DARPin (designed ankyrin repeat proteins) G3 is a small (molecular weight, 14 kDa) scaffold protein with picomolar affinity to HER2. The aim of this first-in-humans study was to evaluate the safety, biodistribution, and dosimetry of ^99m^Tc-(HE)_3_-G3. **Methods:** Three cohorts of patients with primary breast cancer (each including at least 4 patients with HER2-negative and 5 patients with HER2-positive tumors) were injected with 1,000, 2,000, or 3,000 μg of ^99m^Tc-(HE)_3_-G3 (287 ± 170 MBq). Whole-body planar imaging followed by SPECT was performed at 2, 4, 6, and 24 h after injection. Vital signs and possible side effects were monitored during imaging and up to 7 d after injection. **Results:** All injections were well tolerated. No side effects were observed. The results of blood and urine analyses did not differ before and after studies. ^99m^Tc-(HE)_3_-G3 cleared rapidly from the blood. The highest uptake was detected in the kidneys and liver followed by the lungs, breasts, and small intestinal content. The hepatic uptake after injection of 2,000 or 3,000 μg was significantly (*P* < 0.05) lower than the uptake after injection of 1,000 μg. Effective doses did not differ significantly between cohorts (average, 0.011 ± 0.004 mSv/MBq). Tumor–to–contralateral site ratios for HER-positive tumors were significantly (*P* < 0.05) higher than for HER2-negative at 2 and 4 h after injection. **Conclusion:** Imaging of HER2 expression using ^99m^Tc-(HE)_3_-G3 is safe and well tolerated and provides a low absorbed dose burden on patients. This imaging enables discernment of HER2-positive and HER2-negative breast cancer. Phase I study data justify further clinical development of ^99m^Tc-(HE)_3_-G3.

## Visual Abstract

**Figure:**
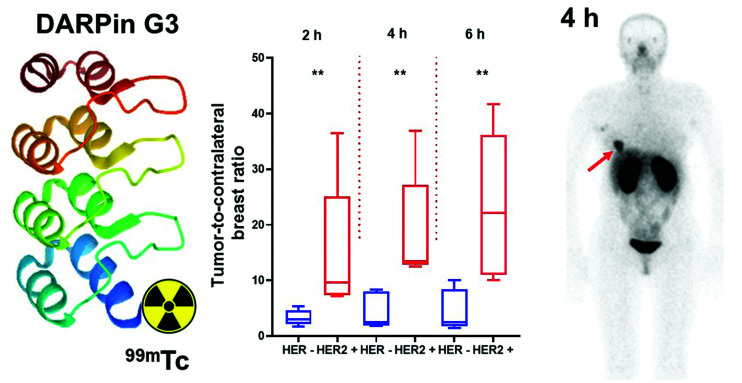

Breast cancer with high levels of human epidermal growth factor receptor type 2 (HER2) expression (3+ immunohistochemistry status) or *HER2* gene amplification (6 or more copies found using in situ hybridization measurements) is clinically defined as HER2-positive (*1*). HER2-positive tumors (15%–20% of total cases) respond to HER2-targeting therapeutics, such as the antibodies trastuzumab and pertuzumab, the tyrosine kinase inhibitor lapatinib, or trastuzumab-based antibody–drug conjugates. Information concerning HER2 expression levels is required for every invasive primary or recurrent breast cancer (*2*) as it is critical for making a decision to use HER2-targeting therapies. To determine HER2 expression in breast cancer, the American Society of Clinical Oncology and College of American Pathologists has recommended biopsy sampling, followed by testing using immunohistochemistry and in situ hybridization in the case of equivocal results from immunohistochemistry tests (*2*).

Although biopsy-based methodology is instrumental in clinical decision making, it is associated with several issues, such as HER2 expression heterogeneity, changing of HER2 expression levels with time, and poor accessibility of some metastases for sampling (*3**,**4*). Since radionuclide molecular imaging provides global information about HER2 expression and is noninvasive, and therefore might be used repeatedly, there is an appreciable interest to develop radiolabeled probes for HER2 visualization (*5**–**7*).

A common approach to imaging HER2 is through the use of therapeutic monoclonal antibodies labeled with long-lived positron emitters, for example, ^89^Zr or ^64^Cu, for PET imaging (*8*). However, the clearance of antibodies from the blood is slow, which is associated with a high background even 4–7 d after injection. Furthermore, harnessing the high sensitivity of PET is essential in this case. This approach is viable in Western Europe and North America, where PET infrastructure is routinely available. However, access to PET is limited in most of the world’s populations living in Africa, Latin America, and Asia. For these regions, a ^99m^Tc-labeled imaging probe would be desirable because SPECT cameras are much more common there.

The use of ^99m^Tc-labeled engineered scaffold proteins for SPECT imaging is a feasible approach for HER2 imaging because these types of imaging probes provide a high contrast only several hours after injection, according to preclinical studies (*9*). Designed ankyrin repeat proteins (DARPins) are one such promising class of engineered scaffold proteins, having small molecular weights (14–18 kDa), high affinity and specificity to selected targets, high chemical and thermal stability, and potentially low production costs (*10*). DARPin G3 binds to HER2 with an affinity of 90 pM and exquisite selectivity (*11*). Goldstein et al. demonstrated that ^111^In-labeled G3 provides specific high-contrast imaging of HER2 in human xenografts in mice 4 h after injection (*12*). For site-specific labeling of G3 with ^99m^Tc, we evaluated the use of ^99m^Tc(CO)_3_^+^ in combination with histidine-containing tags at different positions in the DARPin (*13*). For clinical translation, we selected DARPin (HE)_3_-G3 with the HEHEHE-tag placed at the N terminus. This variant demonstrated significantly (*P* < 0.05) higher tumor-to-liver, tumor-to-muscle, and tumor-to-bone uptake ratios compared with the other tested variants. These higher uptake ratios indicated that the imaging contrast would be higher in the main metastatic sites of breast cancer.

The aim in this first-in-humans study was to evaluate the safety and distribution of ^99m^Tc-(HE)_3_-G3 in patients with primary HER2-positive and HER2-negative breast cancer.

The primary objectives of this study (ClinicalTrials.gov identifier: NCT04277338) were to obtain initial information concerning the safety and tolerability of ^99m^Tc-(HE)_3_-G3 after a single intravenous injection; to assess the distribution of ^99m^Tc-(HE)_3_-G3 in normal tissues and in tumors over time; and to evaluate the dosimetry of ^99m^Tc-(HE)_3_-G3.

The secondary objective was to compare the tumor imaging data with HER2 expression data obtained by immunohistochemistry and fluorescent in situ hybridization (FISH) analysis of biopsy samples.

Because clinical data for other scaffold proteins (*14**,**15*) had demonstrated that the mass of injected protein has a strong influence on its biodistribution and targeting properties, 3 levels of the injected protein mass (1,000, 2,000, and 3,000 μg) were tested.

## MATERIALS AND METHODS

### Patients

This was a prospective, open-label, nonrandomized phase I diagnostic study in patients with untreated primary breast cancer. Both the initial protocol and the further extension of patient cohorts were approved by the Scientific Council of the Cancer Research Institute and Board of Medical Ethics, Tomsk National Research Medical Center of the Russian Academy of Sciences, and all subjects provided written informed consent. Female patients (age, 18–80 y) with HER2 status previously determined using biopsy material from the primary tumor according to the guidelines of the American Society of Clinical Oncology (*2*) were eligible.

According to protocol, patients were divided into 3 cohorts (injected with 1,000, 2,000, or 3,000 μg of ^99m^Tc-(HE)_3_-G3), each including at least 5 patients with HER2-positive and at least 4 patients with HER2-negative tumors. Enrollment into the cohort with higher injected dose was initiated following accomplished safety evaluation for the preceding cohort with lower injected dose. In each cohort, consecutive patients were enrolled until required numbers for each category (HER2-positive and HER2-negative) were obtained.

The inclusion criteria were diagnosis of primary breast cancer with possible lymph node metastases; at least 1 lesion > 1.0 cm in greatest diameter; hematologic, liver, and renal function test results within the normal limits; a negative pregnancy test for patients of childbearing potential; and capability to undergo the diagnostic investigations planned in the study.

The exclusion criteria were other concurrent malignancies; autoimmune disease or history of autoimmune disease; and active infection or history of severe infection. One patient had a previous nephrectomy because of a car accident injury. Although such situations were not listed in the exclusion criteria, the patient was excluded from the study because this might influence the renal elimination rate and result in a nonrepresentative biodistribution pattern.

**FIGURE 1.**
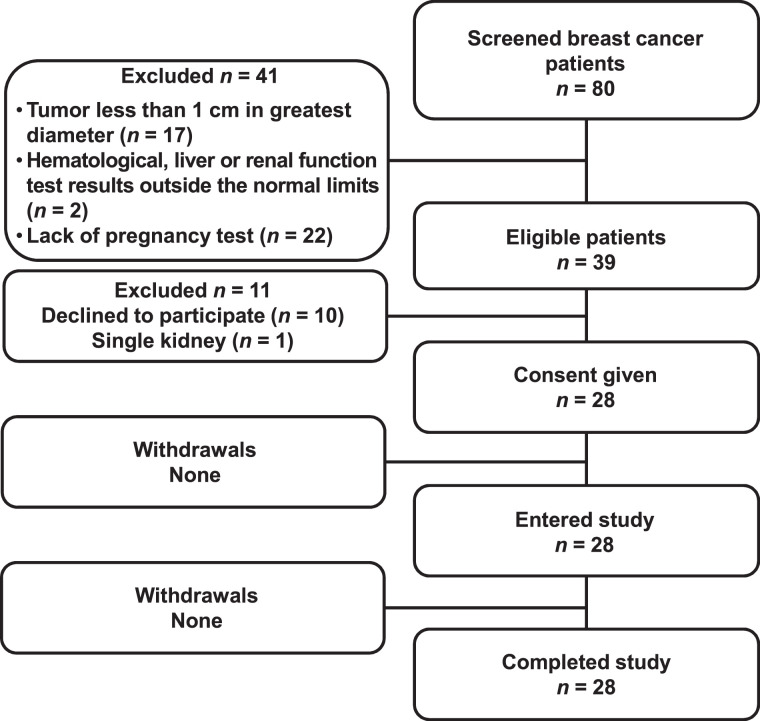
Standards for Reporting of Diagnostic Accuracy Studies (STARD) flow diagram.

Twenty-eight patients were enrolled in the trial (Table 1; Fig. 1).

**TABLE 1.** Patient Characteristics

Patient	Age (y)	HER2 status in primary tumor before imaging (IHC/FISH)	*HER2* gene amplification (determined after imaging)	Primary tumor status (ER/PgR)	Clinical stage before imaging
1,000 μg; mean tumor size, 28 ± 11 mm
1	68	1+ (IHC)	FISH-	ER+/PgR-	IIA (T2N0M0)
2	62	1+ (IHC)	FISH-	ER+/PgR+	I (T1N0M0)
3	66	1+ (IHC)	FISH-	ER+/PgR+	IIA (T2N0M0)
4	48	0 (IHC)	FISH-	ER-/PgR-	IIA (T2N0M0)
5	50	3+ (IHC)	FISH+	ER+/PgR+	IIA (T2N0M0)
6	70	3+ (IHC)	FISH-	ER+/PgR+	IIA (T2N0M0)
7	30	3+ (IHC)	FISH+	ER+/PgR+	IIB (T2N1M0)
8	59	2+ (IHC)/FISH+		ER-/PgR-	IIA (T2N0M0)
9	45	(IHC)3+	FISH-	ER+/PgR-	IIB (T2N1M0)
2,000 μg; mean tumor size, 25 ± 6 mm
10	50	1+ (IHC)	FISH-	ER+/PgR+	IIA (T2N0M0)
11	57	1+ (IHC)	FISH-	ER+/PgR+	I (T1N0M0)
12	43	1+ (IHC)	FISH-	ER+/PgR+	IIA (T2N0M0)
13	51	1+ (IHC)	FISH-	ER-/PgR-	IIA (T2N0M0)
14	65	3+ (IHC)	FISH+	ER+/PgR+	IIA (T2N0M0)
15	35	3+ (IHC)	FISH+	ER+/PgR+	I (T1N0M0)
16	50	1+ (IHC)*	FISH+	ER+/PgR+	IIA (T2N0M0)
17	68	2+ (IHC)/FISH+		ER+/PgR-	IIA (T2N0M0)
18	68	3+ (IHC)	FISH+	ER+/PgR+	IIA (T2N0M0)
3,000 μg; mean tumor size 22 ± 7 mm
19	37	1+ (IHC)	FISH-	ER-/PgR-	IIA (T2N0M0)
20	45	1+ (IHC)	FISH-	ER+/PgR+	IIA (T2N0M0)
21	56	1+ (IHC)	FISH-	ER+/PgR+	IIA (T2N0M0)
22	45	1+ (IHC)	FISH-	ER+/PgR+	IIA (T2N0M0)
23	36	1+ (IHC)	FISH-	ER+/PgR+	IIA (T2N0M0)
24	48	3+ (IHC)	FISH+	ER+/PgR+	IIВ (T2N1M0)
25	58	3+ (IHC)	FISH+	ER+/PgR+	IIA (T2N0M0)
26	47	1+ (IHC)*	FISH+	ER+/PgR+	IIA (T2N0M0)
27	61	3+ (IHC)	FISH+	ER+/PgR-	IV (T4N3M1)
28	49	3+ (IHC)	FISH+	ER+/PgR+	I (T1N0M0)

* Postimaging FISH evaluation demonstrated *HER2* gene amplification.

IHC = immunohistochemistry; ER = estrogen receptor; PgR = progesterone receptor;+ = positive; - = negative.

Mammograms (Giotto Image); biopsy samples; breast, regional lymph nodes, and liver ultrasound images (GE LOGIQ E9); bone scans (Siemens E.Cam 180) using ^99m^Tc-pyrophosphate; and chest CT scans (Siemens Somatom Emotions 16 ECO) were obtained for all patients according to the local standard of care.

The level of HER2 expression in biopsy samples was determined by immunohistochemistry using the Herceptest (DAKO). In tumors with a score of 2+ or in cases of questionable results, HER2 amplification was assessed by FISH using a KBI-10701 probe (Kreatech). To confirm imaging results, FISH analysis was performed after imaging for all patients. The tumors were classified as HER2-positive (HercepTest score 3+ or HercepTest score 2+ and FISH-positive) and HER2-negative (HercepTest score 0 or 1+, or score 2+ but FISH-negative).

### Imaging Protocol

(HE)_3_-G3 was labeled with ^99m^Tc using the protocol reported earlier (*13*). The yield was 83% ± 9% and the radiochemical purity was more than 98%.

^99m^Tc-(HE)_3_-G3 was injected as an intravenous bolus. The injected protein dose was 1,000 μg of (HE)_3_-G3 for patients 1–9, 2,000 μg of (HE)_3_-G3 for patients 10–18, and 3,000 μg of (HE)_3_-G3 for patients 19–29. The average injected activity was 287 ± 170 MBq. A Siemens E.Cam 180 scanner equipped with a high-resolution low-energy collimator was used for imaging. Anterior and posterior whole-body planar imaging (at a scan speed of 12  cm/min, 1,024 × 256 pixel matrix) and SPECT scanning (32 projections, 30 s each, 128 × 128 pixel matrix) were performed at 2, 4, 6, and 24 h. The SPECT data were reconstructed by iterative reconstruction with a gaussian filter and the application of scatter correction using the E.Soft computer system for scintigraphic data processing. Patient 27 was imaged using a Siemens Symbia Intevo Bold scanner equipped with a high-resolution low-energy collimator. Whole-body imaging (at 2, 4, 6, and 24 h) and SPECT scanning (at 4, 6, and 24 h) were performed in the same mode as above. After 2 h, 2 SPECT/CT bed scans were obtained (60 projections, 20 s each, 256 × 256 pixel matrix, 130 kV, effective 36 mAs).

Vital signs and possible side effects were monitored during the investigation (0–24 h after injection) and 3–7 d after the injection. Blood and urine analyses were performed 1 and 3 d after the injection.

### Evaluation of Distribution and Dosimetry

Regions of interest (ROI) were drawn over organs of interest and the body contour on the anterior and posterior whole-body images, and a geometric mean of counts at 2, 4, 6, and 24 h was calculated for each ROI. For quantification, a known activity of ^99m^Tc in a water-filled phantom in combination with Chang’s correction was used. A ROI was placed over the heart to assess the activity in the blood. Data were fitted to single exponential functions, and residence times were calculated as areas under fitted curves using Prism 8 (GraphPad Software, LLC). Absorbed doses were calculated by OLINDA/EXM 1.1 using an adult female phantom.

To calculate tumor–to–contralateral breast and tumor-to-liver ratios, a 3.5-cm^3^ volume of interest was drawn on tomograms centered on the highest tumor uptake, and counts were recorded. Thereafter, this volume of interest was copied to the contralateral breast to obtain reference counts. The tumor–to–contralateral breast ratio for each primary tumor was calculated and matched with the biopsy-based data concerning HER2 expression in the same tumor.

### Statistics

Values are reported as mean ± SD. Differences between uptake in organs at different time points were analyzed using 1-way ANOVA. The nonparametric Mann–Whitney *U* test was used to determine whether the differences between tumor–to–contralateral breast ratios for HER2-positive and HER2-negative tumors were significant. A 2-sided *P* value of less than 0.05 was considered significant.

## RESULTS

### Safety and Tolerability

The intravenous bolus administration of ^99m^Tc-(HE)_3_-G3 was well tolerated by all 29 patients, independent of the injected protein mass. Changes in vital signs or adverse reactions were not registered during imaging or the follow-up period. No relevant changes in blood or urine samples were found after their analyses.

### Evaluation of Distribution and Dosimetry

**FIGURE 2.**
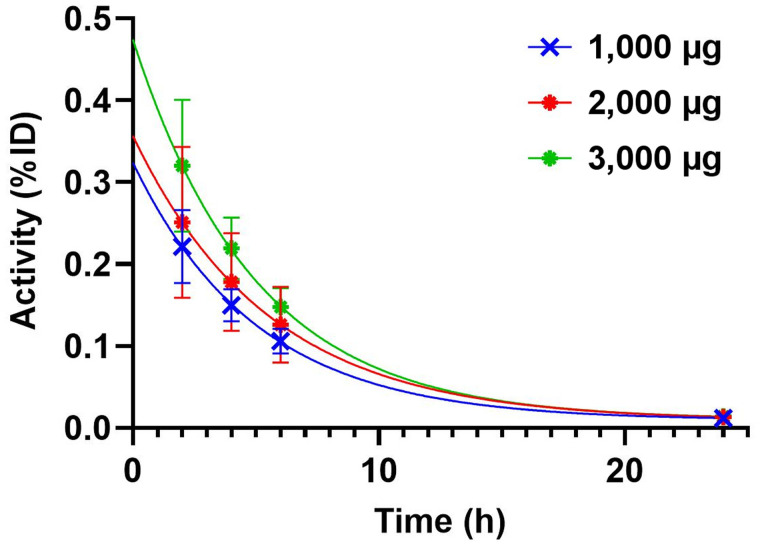
Kinetics of elimination of ^99m^Tc-(HE)_3_-G3 from blood. Data were calculated on the basis of count rates in ROIs placed over hearts.

The kinetics of ^99m^Tc-(HE)_3_-G3 elimination from blood is shown in Figure 2. The elimination half-lives were comparable for all injected protein doses. These elimination half-lives were 3.5 h (95% CI 2.3–6.0 h), 3.8 h (95% CI 3.4–4.3 h), and 3.4 h (95% CI 2.6–4.6 h) for 1,000 μg, 2,000 μg, and 3,000 μg, respectively.

**FIGURE 3.**
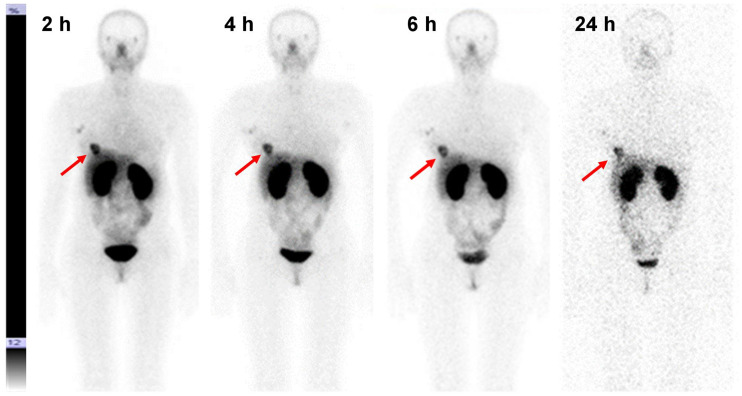
Anterior planar images at 2, 4, 6, and 24 h after injection of 3,000 μg of ^99m^Tc-(HE)_3_-G3 (patient 24). Upper setting of scale window is 12% of maximum counts. Arrows point at lesions.

The kidneys and liver were the organs with the highest uptake of activity (Table 2; Fig. 3). Noticeable activity was also observed in the lungs, small intestines, and lacrimal and salivary glands. Injections with 2,000 and 3,000 μg resulted in a significant (*P* < 0.05) reduction in hepatic uptake compared with 1,000-μg injections. The difference between hepatic uptake after injection with 2,000 and 3,000 μg was not significant, although there was a strong tendency toward reduced uptake with an increase in injected mass (*R*^2^ in the range between 0.86 and 0.95) (Supplemental Fig. 1; supplemental materials are available at http://jnm.snmjournals.org). The decay-corrected uptake in the liver and kidneys did not differ significantly between 2 and 24 h. There was a significant gradual decrease in uptake in the small intestines and lungs between the first and last time points.

**TABLE 2.** Uptake of ^99m^Tc in Tumor-Free Areas of Organs with Highest Uptake on SPECT Images After Injection of ^99m^Tc-(HE)_3_-G3 (Decay Corrected)

	2 h			4 h			6 h			24 h		
Organ	1,000 μg	2,000 μg	3,000 μg	1,000 μg	2,000 μg	3,000 μg	1,000 μg	2,000 μg	3,000 μg	1,000 μg	2,000 μg	3,000 μg
Breast	2.1 ± 0.6	2.4 ± 0.6	2.4 ± 0.6	2.1 ± 0.4	2.0 ± 0.5	2.1 ± 0.5	2.3 ± 1.1	1.8 ± 0.5	1.9 ± 0.3	1.7 ± 0.2	1.7 ± 0.5	1.6 ± 0.4
Small intestines	2.5 ± 0.6	2.4 ± 0.5	2.9 ± 0.6	2.1 ± 0.4	2.2 ± 0.4	2.5 ± 0.6	2.1 ± 0.8	1.9 ± 0.5	2.0 ± 0.4	1.7 ± 0.4	1.7 ± 0.5	1.8 ± 0.6
Kidney	24 ± 5	28 ± 6	29 ± 11	22 ± 5	28 ± 6	29 ± 11	24 ± 5	28 ± 6	29 ± 11	21 ± 5	21 ± 5	26 ± 12
Liver	11 ± 2	6 ± 2*	5 ± 2*	12 ± 4	6 ± 2*	5 ± 2*	10 ± 3	6 ± 2*	4 ± 1*	9 ± 3	5 ± 2*	4 ± 2*
Lungs	2.4 ± 0.6	2.4 ± 0.7	2.7 ± 0.9	2.2 ± 0.5	2.2 ± 0.7	2.2 ± 0.6	1.9 ± 0.6	2.0 ± 0.6	2.0 ± 0.4	1.9 ± 0.5	1.8 ± 0.6	1.6 ± 0.4

* Significant difference in uptake compared with injection of 1,000 μg ^99m^Tc-(HE)_3_-G3.

The evaluation of the absorbed doses is shown in Table 3. The highest absorbed dose was in the kidneys, followed by the adrenals, urinary bladder wall, liver, gallbladder wall, and ovaries. The absorbed dose to the liver was significantly (*P* < 0.05) higher after injection with 1,000 μg than after injection with 2,000 and 3,000 μg. The effective doses were 0.011 ± 0.001, 0.012 ± 0.006, and 0.012 ± 0.003 mSv/MBq for 1,000, 2,000 and 3,000 μg, respectively. An effective dose of 3.2–3.4 mSv would result from the typical injected activity of 250 MBq used in this study.

**TABLE 3.** Absorbed Doses after Injection of 1,000, 2,000, and 3,000 μg of ^99m^Tc-(HE)_3_-G3

	Absorbed dose, mGy/MBq		
Site	1,000 μg	2,000 μg	3,000 μg
Adrenals	0.031 ± 0.007	0.031 ± 0.007	0.032 ± 0.002
Brain	0.0010 ± 0.0004	0.0011 ± 0.0002	0.0012 ± 0.0001
Breasts	0.008 ± 0.002	0.007 ± 0.001	0.008 ± 0.001
Gallbladder wall	0.017 ± 0.003	0.015 ± 0.004	0.014 ± 0.002
LLI wall	0.005 ± 0.001	0.006 ± 0.003	0.006 ± 0.001
Small intestine	0.0076 ± 0.0010	0.009 ± 0.004	0.009 ± 0.002
Stomach wall	0.0060 ± 0.0008	0.006 ± 0.001	0.007 ± 0.002
ULI wall	0.007 ± 0.001	0.008 ± 0.003	0.009 ± 0.002
Heart wall	0.004 ± 0.001	0.004 ± 0.001	0.0042 ± 0.0007
Kidneys	0.10 ± 0.02	0.10 ± 0.03	0.13 ± 0.05
Liver	0.016 ± 0.003	0.011 ± 0.003*	0.0100 ± 0.0008*
Lungs	0.005 ± 0.001	0.005 ± 0.001	0.006 ± 0.001
Muscle	0.0024 ± 0.0005	0.003 ± 0.001	0.0028 ± 0.0007
Ovaries	0.014 ± 0.005	0.014 ± 0.008	0.013 ± 0.003
Pancreas	0.012 ± 0.001	0.013 ± 0.003	0.016 ± 0.004
Red marrow	0.0033 ± 0.0007	0.004 ± 0.001	0.004 ± 0.001
Osteogenic cells	0.006 ± 0.002	0.006 ± 0.002	0.007 ± 0.001
Skin	0.0014 ± 0.0004	0.0015 ± 0.0003	0.0017 ± 0.0003
Spleen	0.010 ± 0.001	0.010 ± 0.003	0.012 ± 0.004
Thymus	0.006 ± 0.001	0.007 ± 0.003	0.0068 ± 0.0002
Thyroid	0.017 ± 0.003	0.018 ± 0.005	0.022 ± 0.003
Urinary bladder wall	0.013 ± 0.007	0.014 ± 0.009	0.019 ± 0.007
Uterus	0.008 ± 0.002	0.055 ± 0.01	0.009 ± 0.003
Total body	0.004 ± 0.001	0.004 ± 0.001	0.004 ± 0.001
Effective dose equivalent (mSv/MBq)	0.017 ± 0.002	0.020 ± 0.012	0.019 ± 0.005
Effective dose (mSv/MBq)	0.011 ± 0.001	0.012 ± 0.006	0.012 ± 0.003

* Significant (*P* < 0.05) difference with absorbed dose after the injection of 1,000 μg ^99m^Tc-(HE)_3_-G3.

LLI = lower large intestine; ULI = upper large intestine.

### Discrimination Between Tumors with High and Low HER2 Expression

**FIGURE 4.**
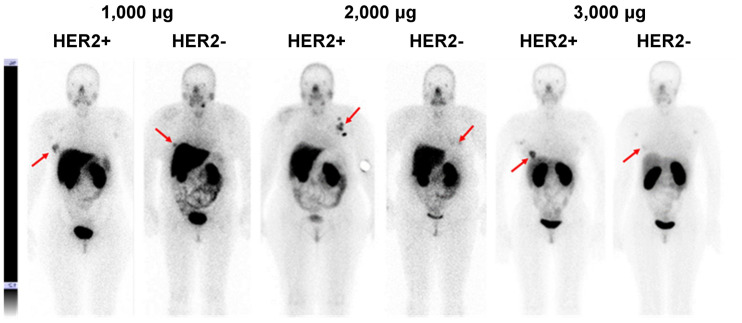
Representative anterior planar images of patients with HER2-positive and HER2-negative tumors 4 h after injections of 1,000, 2,000, or 3,000 μg of ^99m^Tc-(HE)_3_-G3. Upper setting of scale window is same for all images, 12% of maximum count rate. Arrows point at lesions.

Imaging using ^99m^Tc-(HE)_3_-G3 enabled the visualization of all known tumors (including HER2-negative tumors) already 2 h after injection (Figs. 3 and 4). HER2-positive tumors remained visible at all time points, but HER2-negative tumors could not be visualized 24 h after injection. Involved lymph nodes were also visualized in 9 patients. The involvement of lymph nodes was confirmed by histologic analysis after core biopsies (*n* = 2) or cytologic analysis after fine-needle biopsies (*n* = 7).

**FIGURE 5.**
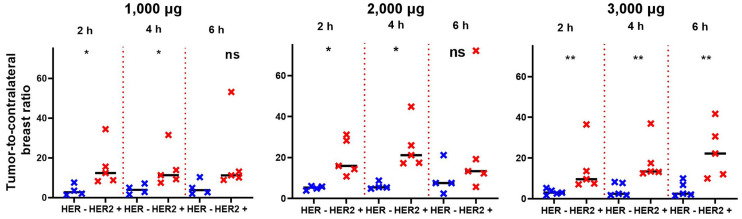
Primary tumor–to–contralateral site ratios after injections of 1,000, 2,000, and 3,000 μg of ^99m^Tc-(HE)_3_-G3. *Marks significant (*P* < 0.05) difference. **Marks highly significant (*P* < 0.01) difference. ns = not significant.

Tumor–to–contralateral site ratios were significantly higher (*P* < 0.05, Mann–Whitney test) for HER2-positive than for HER2-negative tumors at 2 and 4 h after injection with 1,000 and 2,000 μg, but the difference was not significant 6 h after injection (Fig. 5). Tumor–to–contralateral ratios after injection with 3,000 μg were significantly (*P* < 0.05, Mann–Whitney test) higher for HER2-positive tumors at 2, 4, and 6 h after injection (Fig. 5).

**FIGURE 6.**
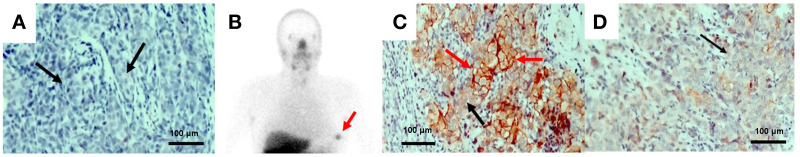
Patient 26. (A) Immunohistochemistry analysis shows very low HER2 expression in biopsy material. (B) Anterior planar image at 4 h after injection; upper setting of scale window is 12% of maximum counts, showing tumor–to–contralateral site ratio typical for HER2-positive tumors. Immunohistochemistry analysis of surgery material shows areas with high (B) and low (C) HER2 expression. Magnification 400×. Black arrows show cells with low and red arrows show cells with high HER2 expression. Arrows point at tumor.

Patient 26 was enrolled in this study as a patient with a HER2-negative tumor based on a 1+ immunohistochemistry score from her core biopsy sample (Fig. 6A). However, the tumor–to–contralateral breast ratio was unusually high (12.5 at 2 h, 3,000-μg dose) for a patient with a HER2-negative lesion (Fig. 6B). On our request, her surgery samples were evaluated, and 35% of the tumor was found to display elevated HER2 expression from immunohistochemistry (Fig. 6C) while the rest of the tumor was HER2-negative (Fig. 6D). On the basis of this finding, trastuzumab treatment was added to her adjuvant therapy. Similarly, a high tumor–to–contralateral site ratio (14.4 at 2 h, 2,000-μg dose) for patient 16 prompted the reevaluation of her biopsy samples using a FISH analysis, which actually suggested a HER2-positive tumor. Further postimaging FISH analysis demonstrated agreement between IHC and FISH data for all other patients except from patient 6, who had HER2 overexpression without gene amplification.

**FIGURE 7.**
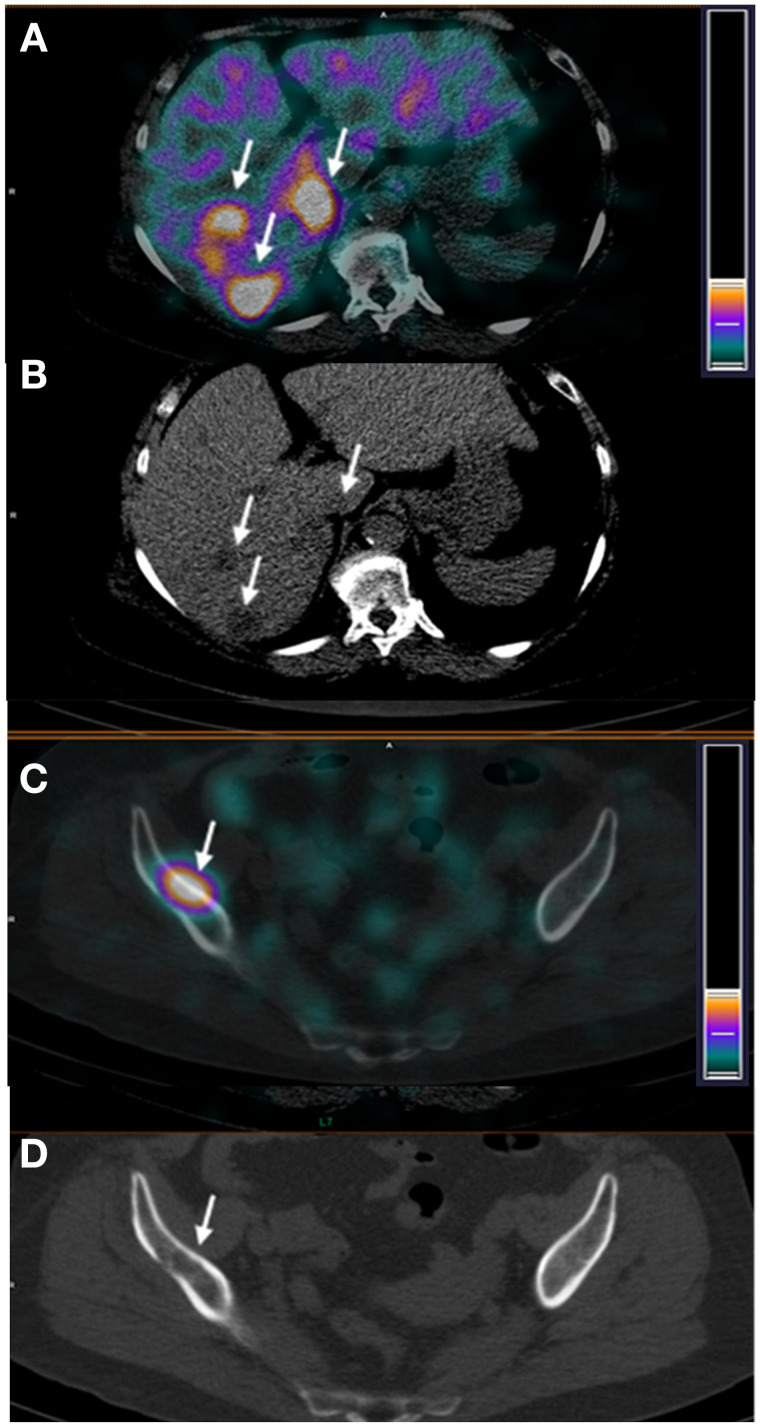
Patient 27. SPECT/CT (A) and CT (B) images of hepatic lesions. SPECT/CT (C) and CT (D) images of iliac bone lesions. Arrows point at lesions.

Patient 27 was enrolled in the study with a large HER2-positive inflammatory breast cancer with axillary node involvement, as well as suspected metastatic sites in the liver. ^99m^Tc-(HE)_3_-G3 imaging demonstrated multiple sites of abnormal accumulation of activity. Presence of metastases in the sites of abnormal accumulation in, for example, the liver (Fig. 7A, tumor–to–reference zone ratio 2.97) and iliac bone (Fig. 7C, tumor–to–reference zone ratio 9.5), was confirmed by diagnostic CT images (Figs. 7B and 7D).

## DISCUSSION

Numerous preclinical studies have demonstrated that small scaffold proteins are promising types of probes for molecular imaging (*16*). Still, only a limited number of such probes have been tested in clinical trials: Affibody molecules (*17*), ADAPTs (*15*), and adnectins (*18*). At the same time, scaffold proteins represents a large variety of molecular forms with different structures, charges and lipophilicity of solvent-exposed amino acids (*19*). These features can substantially modify off-target interactions, impact the biodistribution of these probes, and influence their imaging contrast. Such considerations necessitate the clinical evaluation of different scaffolds to select the most promising ones.

This study demonstrated that the injection of DARPins (up to 3 mg) is well tolerated and not associated with any adverse effects. A combination of rapid systemic clearance and the favorable dosimetry properties of ^99m^Tc ensured a moderate effective dose. In the current study, typical equivalent doses were 3.2–3.4 mSv. However, because the optimal imaging time is between 4 and 6 h, the injected activity could still be reduced at least twice and accordingly further reduce the dose burden to patients. This aspect offers advantages compared with immuno-PET, where the radiation burden is higher by an order of magnitude.

The important features of ^99m^Tc-(HE)_3_-G3 are rapid localization in tumors and quick clearance from the blood (Fig. 3). Both of these features might be explained by the small size of this targeting probe and they enabled the clear visualization of tumors as early as 2 h after injection (Fig. 3). It should also be noted that cells in clinically defined HER2-negative tumors (immunohistochemistry score of 2+ and FISH-negative) might still express hundreds of thousands of HER2 receptors per cell (*20*). Accordingly, HER2-negative tumors were also visualized (Fig. 4). However, tumor–to–contralateral breast ratios were significantly (*P* < 0.05, Mann–Whitney test) higher for HER2-positive lesions at 2 and 4 h in 1,000-μg and 2,000-μg cohorts, and at 2, 4, and 6 h in the 3,000-μg cohort (Fig. 5). Such a simple approach is feasible even in developing countries as it does not require regular exact calibration of SPECT cameras. Unexpectedly, HER2 imaging using ^99m^Tc-(HE)_3_-G3 has already proved its worth in this study. High tumor–to–contralateral breast ratios triggered the reevaluation of HER2 status for patients 16 and 26, who were enrolled in this trial as having HER2-negative tumors. In both cases, a HER2-positive status has been confirmed, indicating that radionuclide molecular imaging can overcome the limitations of biopsy-based methods caused by heterogeneous target expression and associated sampling errors.

The aspect of injected mass was found essential in earlier studies with Affibody molecules (*14**,**17*) and ADAPT6 (*15*). When a low protein mass is injected (100 μg for Affibody molecules or 250 μg for ADAPT6 in the case of HER2 imaging), the binding to HER2 expressed on hepatocytes results in a high liver uptake and sequestration of the probe from circulation. This prevents delivery of the radionuclide to tumors and increases the background during the imaging of liver metastases, which are common in breast cancer. The current study demonstrated that optimization of the injected mass is essential for DARPin-based HER2 imaging probes as well. An increase of the injected mass from 1,000 to 3,000 μg decreased hepatic uptake by more than half (Table 2; Supplemental Fig. 1). This decrease of hepatic uptake created a precondition for the clear visualization of HER2-positive liver metastases in patient 27 (Fig. 7). The example of patient 27 indicates that ^99m^Tc-(HE)_3_-G3 has the capability to enable visualization of metastases in multiple locations, including the bone and liver. Ultimately, the tracer should be used for detection of HER2 expression in metastatic breast cancer. Such findings during the phase I trial motivate its further development.

## CONCLUSION

Injections of ^99m^Tc-(HE)_3_-G3 are safe and result in low absorbed and effective doses. Preliminary data suggest that SPECT/CT using ^99m^Tc-(HE)_3_-G3 could distinguish HER2-positive from HER2-negative primary breast cancer. An injected protein mass between 2,000 and 3,000 μg is desirable to suppress hepatic uptake. Further clinical development of ^99m^Tc-(HE)_3_-G3 for imaging of HER2 expression in cancers is warranted.

## Notes

KEY POINTS **QUESTION:** Is the use of ^99m^Tc-(HE)_3_-G3 for HER2 imaging tolerable, safe, and informative? **PERTINENT FINDINGS:** ^99m^Tc-(HE)_3_-G3 is safe, is associated with low dose burdens, and enables the differentiation of HER2-positive and HER2-negative primary breast cancer lesions at 2–4 h after injection. **IMPLICATION FOR PATIENT CARE:** Results from this study suggest that further clinical development of ^99m^Tc-(HE)_3_-G3 might provide a probe for the SPECT imaging of HER2 expression.
